# Quantitative Determination of Fluorine Content in Blends of Polylactide (PLA)–Talc Using Near Infrared Spectroscopy

**DOI:** 10.3390/s16081216

**Published:** 2016-08-02

**Authors:** Elena Tamburini, Chiara Tagliati, Tiziano Bonato, Stefania Costa, Chiara Scapoli, Paola Pedrini

**Affiliations:** 1 Department of Life Science and Biotechnology, University of Ferrara, Via L. Borsari, Ferrara 46 | 44121, Italy; stefania.costa@unife.it (S.C.); scc@unife.it (C.S.); pdp@unife.it (P.P.); 2 Lab Control Srl, Chemical Analysis and Technological Services, Via Cà Donà, San Martino di Venezze, Rovigo 545 | 45030, Italy; chiara.tagliati@student.unife.it (C.T.); ambiente@lab-control.it (T.B.)

**Keywords:** PLA, polylactide, talc, fluorine, blends, near infrared spectroscopy, quantitative calibration

## Abstract

Near-infrared spectroscopy (NIRS) has been widely used for quantitative and/or qualitative determination of a wide range of matrices. The objective of this study was to develop a NIRS method for the quantitative determination of fluorine content in polylactide (PLA)-talc blends. A blending profile was obtained by mixing different amounts of PLA granules and talc powder. The calibration model was built correlating wet chemical data (alkali digestion method) and NIR spectra. Using FT (Fourier Transform)-NIR technique, a Partial Least Squares (PLS) regression model was set-up, in a concentration interval of 0 ppm of pure PLA to 800 ppm of pure talc. Fluorine content prediction (R^2^_cal_ = 0.9498; standard error of calibration, SEC = 34.77; standard error of cross-validation, SECV = 46.94) was then externally validated by means of a further 15 independent samples (R^2^_EX.V_ = 0.8955; root mean standard error of prediction, RMSEP = 61.08). A positive relationship between an inorganic component as fluorine and NIR signal has been evidenced, and used to obtain quantitative analytical information from the spectra.

## 1. Introduction

Today, polymers are principally of non-renewable origin, and based on crude oil and natural gas resources. Whereas some of them are recycled and reused, the majority are still disposed of in landfills or discharged in the environment [1]. Over the last years, several sustained research have been carried out on biodegradable polymers derived from renewable sources as one of the solutions to reduce waste disposal problems and to limit the dependence on petroleum-based materials [2]. From a chemical point of view, polylactide or polylactic acid (PLA) is a polyester usually made from α-hydroxy acids, and is considered biodegradable and compostable [3]. The monomer of PLA, lactic acid (2-hydroxy propionic acid), is principally produced by the fermentation of renewable resources such as starch and sugars, and is nowadays only rarely obtained from petroleum-based ethylene [4]. PLA is produced commercially through the synthesis of lactide (a cyclic ester composed of two lactic acid molecules), followed by a polycondensation of the ring-open lactides. Even though lactic acid can exist as d- or l-enantiomer, the well-known stereoselectivity of microbial processes leads almost exclusively to l-Lactic acid, and subsequent optical inversion is possible. It is worth noting that based on the proportion of the two enantiomers in the polymer chain, different properties of PLA can be obtained, depending on the performance required [5]. In fact, PLA is one of the most widely used biopolymers, especially in biomedical and packaging applications [6]. PLA has several excellent properties, such as low toxicity, good transparency, and high resistance; however, compared with other conventional polymers, it still shows lower technical performance—particularly with respect to mechanical properties and slow crystallization rate [7]. Several different technological improvements have been attempted to improve the properties of PLA; for example, blending PLA with other materials, and the use fillers or nanofillers as additives [8,9]. Conventional inorganic fillers (e.g., talc) are still widely used as reinforcing and nucleating agents in other diffused polymers, because they have the potential to assure relevant and low-cost improvements in formulations [10]. To obtain a significant decrease in processing time and a simultaneous increase in crystallinity, 2%–5% talc can be added to PLA [11]. Talc is a hydrated magnesium silicate, with the general formula Mg_3_SiO_10_(OH)_2_. However, talc structure also contains several other ions in smaller quantity or traces, among which fluorine (F) deserves particular attention due to its high toxicity [12], which can affect the overall compostability of PLA manufacts. For this reason, the current regulations on composting and biodegradability fixes strict limits on the fluorine content of biopolymer materials [13], making analytical controls on products destined for the market mandatory. The official chemical assay is based on the use of fluorine calorimetric bomb (FCB) under oxygen pressure, followed by ion chromatography analysis [14]. Alkali fusion digestion can also be used, treating samples with a concentrated solution of sodium hydroxide at high temperature [15]. FBC involves nearly explosive reactions at high pressures, long and laborious procedures, and often gives underestimated quantification [16]. Near Infrared Spectroscopy (NIRS) could represent a promising alternative technique for fluorine quantification. In recent decades, NIRS has been becoming particularly popular for industrial applications because it can rapidly and non-destructively analyse samples with minimum preparation [17]. The ability of NIRS to analyse the chemical properties of samples relies on the selective absorption of light (in the range 800–2500 nm) by the overtones and combinations of vibrations of polar bonds (i.e., CO, OH, CH and NH), which have their fundamental vibrations in the mid-infrared region [18]. Several studies have described how it can be used in combination with multivariate calibration techniques for the quantitative prediction of parameters that are of interest to the polymers industry [19,20,21]. In this study, a quantitative Fourier Transform-NIRS (FT-NIRS) method for fluorine detection in PLA—with increasing loadings of talc before melting—has been set up. Despite talc’s inorganic nature, NIRS has been demonstrated to be a powerful tool in the characterization of its chemical composition (and of many other hydroxylated minerals), due to the possibility of recognizing the presence of OH groups in the octahedral crystal structure around the elements, via hydroxyl bond vibrations [22]. A Partial Least Squares Regression (PLSR) model has been built from samples at different concentrations of fluorine, artificially constructed in laboratory in order to obtain a sufficiently wide and representative calibration range.

## 2. Materials and Methods

*Polylactic acid (PLA)–talc blends*. A known amount of pure PLA granules were mixed with a known amount of talc powder. PLA and talc were supplied by an industrial manufacturer and were derived from its own production plant. Talc powder was previously analysed with a wet chemical method to determine the concentration of fluorine (see below). Thirteen different blends were created, increasing the amount of talc (0%–100%) and decreasing the amount of PLA granules (100%–0%), so as to maintain constant the final weight at 3.53 ± 0.05 g at room temperature. In such a way, the concentration of fluorine in samples varies in the interval 0–800 ppm. Samples were well mixed using a lab-scale powder mixing system (BT200 Batch Homogenizer, Dynaken, Silangor, Malaysia) and then submitted to NIR analysis.

*Wet chemical fluorine assays*. The alkali digestion method was used [23]. An aliquot of talc sample was ground, homogenized, and incinerated at 550 °C. Two grams of ash were digested at high temperature for 2 h with 50 mL of NaOH 30% w/v. Sample volume was reported at 50 mL with water, filtered on cellulose acetate, and then analysed on an ion chromatography system 930 Compact IC Flex (Metrohm, Herisau, Switzerland). Samples were separated on an anion-exchange column (250 mm × 4.0 mm i.d.), using Na_2_CO_3_ 0.27 M and NaHCO_3_ 1.00 M as eluents, at a flow rate of 1.2 mL/min at room temperature. The injection volume was 25 μL.

Water for preparation of all solutions was deionized by a Milli-Q purification system with a 0.2 μm fiber filter (Millipore, Billerica, MA, USA). All reagents were ultrapure and of HPLC grade (Sigma Aldrich, St. Louis, MO, USA).

*FT-NIR spectra collection*. FT-NIR diffuse reflectance spectra of samples were collected with a NIRFLex N-500 (Büchi, Flawil, Switzerland), equipped with the Solids Cell Module (Büchi, Switzerland) designed for standard Petri dishes (Schott, Mainz, Germany), and set up with a polarization interferometer with TeO_2_ wedges. Fresh samples were flattened on the glass surface of a 9.0 cm-diameter standard cup by using a stainless steel load disk. Due to the eccentrically rotating cup housing, two scans for each sample were taken. The instrument was designed to be operational for working temperature conditions from 5 up to 35 °C, without any drift of the spectra signal. The reflectance spectra were recorded using NIRWare 1.4 (Büchi, Switzerland) and scanning the full range, from 10,000 to 4000 cm^−1^, at 8 cm^−1^ intervals; measurements were carried out at 2–4 scans/s with a wavenumber accuracy of ±0.2 cm^–1^ (measured with HF gas cell at an ambient temperature of 25 ± 5 °C). To obtain a good signal-to-noise ratio, 128 scans for each spectrum were averaged during each spectral acquisition, resulting in a total measurement time of 30 s. Every spectrum acquisition was preceded by the acquisition of an internal reference to optimize the spectrum baseline.

*Chemometric analysis*. All chemometric analysis, including calibration and validation, were performed using NIRCal 5.0 (Buchi, Switzerland). The raw optical data were pre-processed with a Standard Normal Variate (SNV) transformation to correct the multiplicative interferences of scatter and particle size, generally found in the reflectance spectra of solid samples [24]. The wavenumbers for each set of processing data were suggested by the NIRCal 5.0, based on the calculation of all regression coefficients between each wavenumber of all the calibration set spectra and the property value. To establish the relationship between the off-line wet chemical data and NIR values, the Partial Least Squares Regression (PLSR) was used. The optimum number of factors to be used was determined by the predicted residual error sum of squares (PRESS) calculation, which shows the sum of squares of deviation between predicted and reference values [25]. The selection of the best quantitative regression models was carried out using squared Pearson correlation coefficient for calibration (R^2^_cal_) and cross validation (R^2^_CV_), standard error of calibration (SEC) and standard error of cross-validation (SECV). Relative Prediction Deviation (RPD)—i.e., the relationship between the SD (Standard Deviation) of the entire population divided by the SEC—was also calculated for both calibrations and cross validations [26].

Permutation random test on PLS regressions was carried out using the lmPerm library in the software RPackage 3.3.1 x Windows [27].

*Cross validation*. Cross validation, as default software output, was performed in blockwise mode, splitting the calibration set into six-fold segments, and testing one segment as a validation set and the remaining as a calibration set. Quality of calibration was described in terms of the Q-value, calculated by the NIRCal 5.0 software combining all relevant statistical measures (SEC, determination coefficients). This quality index qualifies the calibrations using a number between 0 (useless) and 1 (ideal). When achieving a Q-value greater than 0.50, the calibration will give reliable results [28]. Outlier detection was performed by the software based on Mahalanobis distance criterion [29]. To test the probability of autocorrelation between time-series spectra, the Durbin–Watson (DW) test, calculated on residuals of PLS regression, was used [30].

*External validation*. The calibration for fluorine was then validated by means of external validation: 15 new independent samples were acquired to obtain additional data and evaluate the predictive capability of the model. The prediction accuracy was considered in terms of squared correlation coefficient (R^2^_EX.V_) and root mean standard error of prediction between predictions and reference values (RMSEP). Finally, the standard error of the laboratory (SEL)—i.e., the error of the reference data—was reported in order to allow a comparison between that value and the NIR performance (SEC and RMSEP).

## 3. Results and Discussion

### 3.1. NIR Spectra and Data Pretreatments

**Figure 1:**
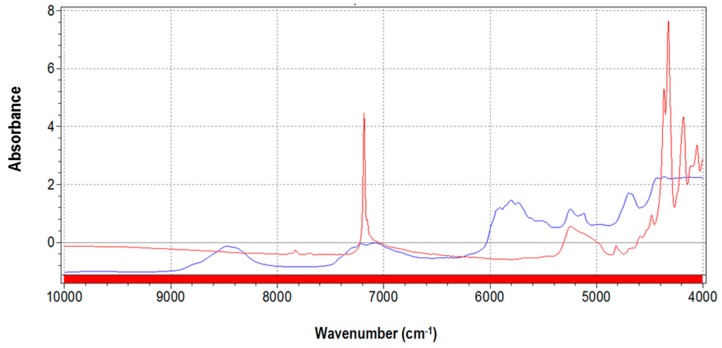
Average spectra of pure talc (red) and of pure polylactide (PLA) polymer (blue).

Average spectra of pure talc containing fluorine used for the blends and pure PLA are shown in Figure 1.

Pure talc shows the typical sharp peak at 7185 cm^−1^ due to fluorine content [30], whilst the PLA spectrum is principally characterized by absorptions at 8400 cm^−1^ and in the interval 6000–4700 cm^−1^ [31].

**Figure 2:**
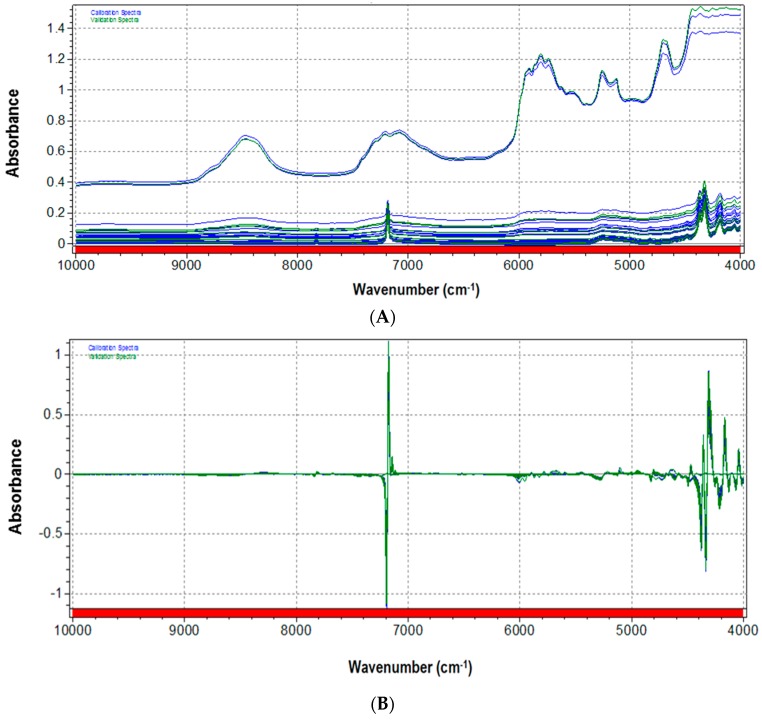
(**A**) Original and (**B**) pretreated spectra of PLA–talc blend samples (at different ratios of PLA/talc content) used in this application.

Three spectra acquisitions for each talc–PLA blend sample have been carried out, for a total of 39 spectra. Original spectra are depicted in Figure 2A. As expected, by comparison with pure components, two main regions could be recognized: in the 7200–6800 cm^-1^ interval, principally corresponding to talc signals; and in the 6000–4700 cm^−1^ interval, mainly influenced by PLA absorptions. Figure 2B shows the same set of spectra after mathematical pretreatment. Raw spectra were pretreated with a combination of Standard Normal Variate (SNV) and first derivative (Savitzky-Golay five points) as mathematical pretreatments. SNV is a mathematical function applied to spectra with the aim of reducing slope variation and minimizing scattering dispersion [32].

SNV removes the multiplicative interferences of scatter, particle size, and the change of light distance. It corrects both multiplicative and additive scatter effects. To remove slope variations on an individual spectrum basis, each element is transformed independently using the calculation suggested by Brereton [33].

First derivative eliminates baseline drifts and small spectral differences are enhanced. Derivatives in general are mainly used to resolve peak overlap (or enhance resolution) and eliminate constant and linear baseline drift between samples, but at the expense of the signal-to-noise ratio, which usually increases. Spectral first derivative has been calculated here by Savitzky-Golay polynomial fitting, where the data within a moving window are fitted by a polynomial of a given degree in order to generate a differential of a chosen degree. In this procedure, it is very important to select the proper differentiation width of the moving window in the function. The width should not exceed one point five times the half width of absorbance peak in the spectra [34].

The final choice of SNV followed by first derivative calculation was the result of trial and error, as the best combination between highlighting spectral information and reducing noise.

**Figure 3:**
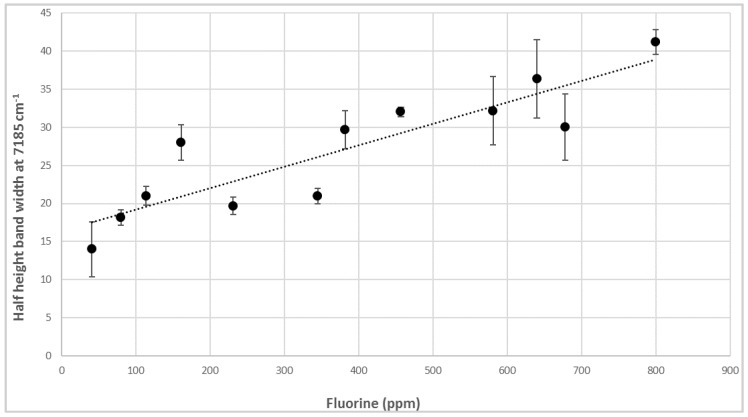
Correlation between half height band width at 7185 cm^−1^ and fluorine content in samples of PLA–talc blends.

NIR spectra are dominated by the first OH-overtone region around 7200–7100 cm^−1^. In particular, the prominent bands at 7185 cm^−1^ and the others at 7156, 7118, and 7073 cm^−1^ derived from talc distinctive signals, due to the OH-stretching vibrations of Mg_3_OH, Mg_2_FeOH, MgFe_2_OH, and Fe_3_OH, respectively [35]. The general crystal structure of talc is a trioctahedral sheet, where each hydroxyl group is bonded to three cations; therefore, the OH-stretching absorption bands are affected by the nature of these cations [36]. The 7185 cm^−1^ half height bandwidths could be correlated with the fluorine content, which enters in the structure as substituting for OH. Band broadening has already been proven to be related to the degree of distortion of the crystal structure induced by the presence of extraneous elements. Here, this trend can be explained by a change in charge distribution within the octahedral structure due to the different electron-withdrawing strengths of F and OH, which modifies the inductive effect and generates an overall structure disorder [37]. A linear correlation between the number of fluorine atoms per unit crystal cell of talc and the half height width of the Mg_3_OH band at 7185 cm^−1^ has been proposed by Petit et al. [22]. Measuring half height bandwidths at 7185 cm^−1^ in our set of spectra corresponding to PLA-talc blends, a quite-good linear correlation has been confirmed (R^2^ = 0.785) (Figure 3), demonstrating that a positive relationship between an inorganic component (e.g., fluorine) and NIR signal exists, and could be properly exploited to obtain quantitative analytical information from the spectra.

In the PLA region, the bands at 5915 and 5785 cm^−1^ can be assigned to the first overtones of asymmetric and symmetric stretching vibrations of CH_3_, respectively [38]. The band at 5495 cm^−1^ is assigned to the first overtone of the CH stretching vibrations, whereas the shoulder at 5680 cm^−1^ can be due to the combination band of asymmetric and symmetric stretching vibrations of the CH_3_ groups. A band corresponding to the second overtones of the C=O stretching vibrations is observed at 5210 cm^−1^. Two overlapped bands at 4750 and 4700 cm^−1^ are associated with the terminal OH groups, as reported by Shinzawa et al. [39].

### 3.2. NIR Calibration and Validation

**Figure 4:**
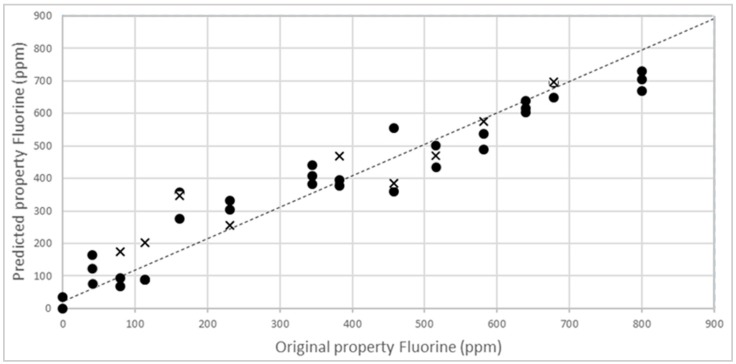
NIR calibration (●) and validation (×) curves for fluorine content in PLA–talc blends.

In the samples used for calibration, fluorine content ranged between 0 ppm in the pure PLA sample, and 800 ppm in the pure talc sample. No outliers were found in the sample set. In blockwise cross validation, all samples were used as the calibration set for quantitative calibrations except for a small group, which is left out. Validation is accomplished by predicting the left out samples and by systematically varying the selection of left out samples. The blockwise internal cross-validation was performed by dividing the entire set in blocks of six spectra and randomly assigning four spectra to the calibration set (C-set) and the remaining two to the validation set (V-set). The calibration curve obtained with the PLS regression model (four factors), and the cross-validation curve are shown in Figure 4. SEC = 34.77 and R^2^ = 0.9498, and SECV = 46.94 and R^2^ = 0.9247 were respectively obtained as statistics for calibration and validation (Table 1). No autocorrelation has been evidenced by the DW statistics, and the predictive ability of the model appeared confirmed by the RPD values.

As expected, the error of NIR prediction (SEC) is higher than the error of reference data (SEL). NIR is a secondary technique on which always burdens errors of the primary technique (reference data). Otherwise, the rapidity of NIR analysis is worth mentioning in comparison with alkali digestion and chromatography, and the complete removal of reagents has to be considered in the overall balance.

Owing to the small data set, a permutation test with 1000 random rearrangements has been carried out on y-vector of NIR predicted data. The empirical distribution of the regression coefficients given from permutations N was centered in −0.0013, with a standard deviation, 0.1501), in permutations tests, the null hypothesis is defined as: the labels assigning samples to classes are interchangeable [40]. Significantly, low *p*-values indicate that the labels are not interchangeable and that the original label configuration is relevant with respect to the data. Performing all possible permutations, and computing the fraction of permutation values that are at least as extreme as the test statistic obtained from the unpermuted data, the level of significance was *p* < 0.00001.

**Figure 5:**
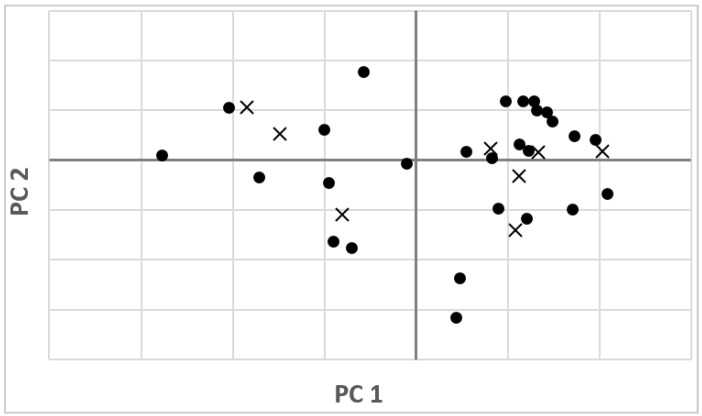
Score plot of the first and second principal components (PCs) of pretreated spectra, in calibration (●) and validation (×) sets

The interpretation of the results of the calibration model is usually carried out by visualizing the score plot. Figure 5 shows the two dimensional (2D) principal components (PCs) score plot using the first two score vectors (PC1 and PC2), derived from pretreated spectra of the samples. The initial two factors, which account for the most spectral variation, 99.48% (98.99% and 0.48% for the first two principal components, PC1 and PC2, respectively). The score plot depicts the distribution between samples within the C-set and V-set for fluorine concentration (ppm).

**Figure 6:**
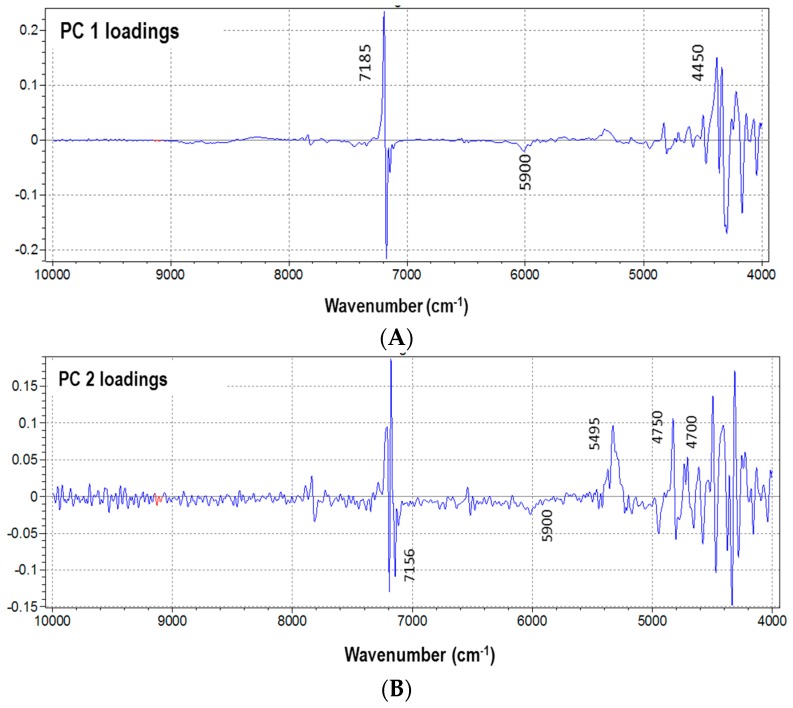
Wavelengths selection from loadings of the first two PCs derived from PLS: (**A**) PC 1 loadings and (**B**) PC 2 loadings.

The loadings resulting from PLS were considered as an indication of the effective wavenumbers that were responsible for the specific features that appeared in the corresponding scores and contributed to the quantification of the property of interest. Figure 6 shows that wavenumbers at 7185 cm^−1^, corresponding to fluorine, and in the range 5900–4500 nm, related to PLA, have the strongest effect on the model, because they bring the most analytical information.

### 3.3. External Validation of the Model

**Figure 7:**
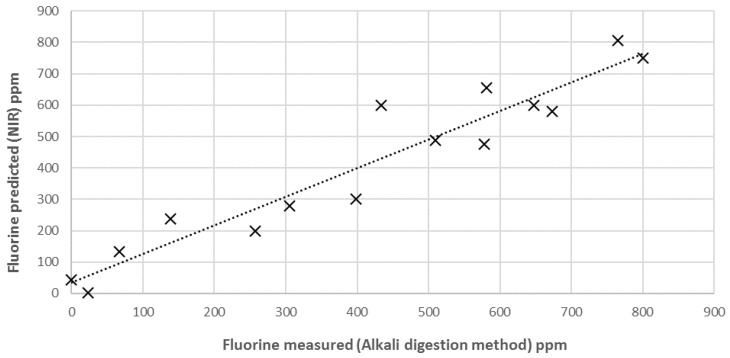
External validation of NIR calibration model for the prediction of fluorine content in unknown samples of PLA-talc blends.

To obtain a reliable model to be applied with unknown samples, the external validation step is fundamental. In fact, the cross-validation procedure is not sufficient to prove the predictive ability and the robustness of the calibration, especially when multi-components and solid samples have to be analysed. Matrix effects, structural complexity and non-homogeneous samples surface, sample composition, and low concentration of parameter of interest could strongly influence the model performance. External validation has to demonstrate the performance of the chosen model for future (unknown) samples, using an independent validation set consisting of samples that have not be used in the creation of the calibration [41]. Accordingly, 15 other new PLA-talc blend samples were created, as described above, and NIR spectra acquired and then analysed with the reference analytical method. In Figure 7, external validation results are shown in terms of NIR-predicted fluorine values vs. concentration obtained with alkali digestion method.

Statistical parameters (R^2^_EX.V_ = 0.8955 and RMSEP = 61.08) have validated the NIR technique to be used for fluorine analysis in PLA–talc blends (Table 2).

## 4. Conclusions

NIR spectra of PLA and talc blends before melting clearly evidenced the presence of both components. In particular, fluorine occurrence in talc generated variations on spectra that can account for measured band broadening. Chemometric results have demonstrated that NIRS could be a suitable tool to rapidly and accurately predict fluorine concentration in PLA-talc blends, avoiding traditional labour-intensive and hazardous methods. The step forward will surely be to apply this technique to PLA manufacts, so as to develop a robust analytical method to measure fluorine content in each sample, in real-time, without destroying it.

## Figures and Tables

**Table 1:** Calibration and cross validation results for fluorine. C-set: calibration set; DW: Durbin–Watson; NIR: near infrared; RPD_cal_: relative prediction deviation of calibration; RPD_CV_: relative prediction deviation of cross-validation; SD: standard deviation; SEC: standard error of calibration; SECV: standard error of cross-validation; SEL: standard error of the laboratory; WN: wavenumber.

Parameter	Fluorine
Units	ppm
SEL-reproducibility	17.34
#samples	39
Outliers	0
Min	0.00
Mean	346.92
Max	800.00
SD	260.17
WN range/step (cm^−1^)	9000–4500/8
Pretreatments	SNV/D1
Regression method	PLS
Number of factors	4
SEC	86.14
R^2^_cal_	0.9471
SECV	76.83
R^2^_CV_	0.9464
NIR repeatability	0.20
DW	2.29
C-Set Durbin–Watson in range 1.5 to 2.5?	yes
Q-value	0.85
RPD_cal_	2.62
RPD_CV_	3.60

**Table 2:** Statistics of validation during external tests for fluorine. RMSEP: root mean standard error of prediction; V-set: validation set.

Parameter	Fluorine
Units	ppm
#samples	15
Outliers	0
Min	0.00
Mean	413.20
Max	800.00
SD	258.46
RMSEP	61.08
R^2^_EX.V_	0.8955
NIR repeatability	0.20
Bias	0.05
Intercept	37.46
Slope	0.85
DW	1.84
V-Set Durbin-Watson in range 1.5 to 2.5?	yes
